# Prognostic utility of the revised post-cardiac arrest syndrome for therapeutic hypothermia score in pediatric patients with post-cardiac arrest syndrome

**DOI:** 10.1016/j.resplu.2026.101489

**Published:** 2026-09-11

**Authors:** Yuta Suenaga, Satoshi Nakano, Takehiro Nitsu, Ikuya Ueta

**Affiliations:** Division of Critical Care Medicine, Saitama Children’s Medical Center, 1-2 Shintoshin, Chuo-ku, Saitama 330-8777, Japan

**Keywords:** Pediatric intensive care unit, Post-cardiac arrest syndrome, rCAST score

## Abstract

**Background:**

The accurate prognosis of post-cardiac arrest syndrome (PCAS) is critical for guiding intensive care and family communication. The revised post-Cardiac Arrest Syndrome for Therapeutic hypothermia (rCAST) score incorporates six readily available clinical variables and has shown promise in children. However, prior validations involved older children. We aimed to externally validate rCAST scores in a cohort of infants and young children.

**Methods:**

We retrospectively examined patients with PCAS (with pre-arrest pediatric cerebral performance category scores of 1–3 and receiving targeted temperature management) admitted to a pediatric intensive care unit (PICU) between December 2016 and March 2024. Patients were stratified into low-, moderate-, and high-severity groups based on their rCAST scores. The primary outcome was neurological status at PICU discharge.

**Results:**

Among 5456 PICU admissions, 75 were included (median age, 17 months [interquartile range: 4–72]; 68% without pre-existing conditions). Favorable neurological outcomes occurred in 28% of patients overall, with 76% in the low (*n* = 17) and 73% in the moderate (*n* = 11) groups, but none in the high group (*n* = 47). The overall in-hospital mortality rate was 60%: 6% in the low group and 87% in the high group (*P* < 0.001). The rCAST score showed good discrimination for both unfavorable neurological outcome (area under the curve [AUC], 0.947; 95% confidence interval [CI], 0.894–1.000) and mortality (AUC, 0.857; 95% CI, 0.750–0.964).

**Conclusions:**

The rCAST score demonstrated good discriminative ability for neurological outcomes in infants and young children with PCAS. However, unfavorable outcomes in some patients with low scores warrant caution in clinical decision-making.

## Highlights

• We externally validated rCAST in infants and young children with PCAS after OHCA.
• rCAST showed good discrimination for neurological outcome and in-hospital mortality.
• Infants and young children were frequently classified as moderate or high severity.
• Unfavorable outcomes in some low-scoring patients warrant clinical caution.

## Background

Pediatric out-of-hospital cardiac arrest (OHCA) is uncommon, occurring in 1–20 per 100,000 children, yet it remains a major public health concern due to its high mortality and poor neurological outcomes.^1^ Survival to hospital discharge is limited, and long-term neurobehavioral outcomes are typically unfavorable. Furthermore, accurate prediction of neurological outcomes in pediatric patients with post-cardiac arrest syndrome (PCAS) is crucial for guiding post-admission management and supporting communication with families; however, reliable prognostic tools remain limited.

Recently, other methods, such as electroencephalography (EEG)^2^ and machine learning-based prediction models,^3^, ^4^ have been explored for prognostication in pediatric PCAS, but these approaches often require specialized equipment or complex data processing. In contrast, clinical scoring systems based on routinely available variables offer greater accessibility and scalability.

The revised post-Cardiac Arrest Syndrome for Therapeutic hypothermia (rCAST) score was developed to predict neurological outcomes in adult patients with PCAS undergoing targeted temperature management (TTM).^5^ The score is calculated using six readily available clinical variables: initial rhythm, witness status, time to return of spontaneous circulation (ROSC), blood pH, blood lactate concentration, and motor score on the Glasgow Coma Scale (GCS). Based on the total score, patients are categorized into low-, moderate-, or high-severity groups. Furthermore, recent external validation studies have demonstrated that the rCAST has high predictive accuracy for neurological outcomes in pediatric patients with PCAS.^6^

Pediatric OHCA predominantly occurs in infants and toddlers.^7^, ^8^, ^9^, ^10^ However, a pediatric rCAST study reported a median age of onset of 8 years,^6^ raising concerns that existing rCAST validations may not adequately represent younger pediatric populations. Therefore, we aimed to externally validate the rCAST score in predicting neurological outcomes in pediatric patients with PCAS following OHCA, including infants and young children. To our knowledge, this is the first study to assess the applicability of the rCAST score in a cohort predominantly consisting of very young pediatric patients.

## Methods

### Study design and participants

This single-center retrospective cohort study was performed at the Division of Critical Care Medicine, Saitama Children’s Medical Center (SCMC), between December 2016 and March 2024. This study was reported in accordance with the TRIPOD+AI reporting guideline.^11^ The study was approved by the Ethical Review Board of the SCMC (approval number: 2024-03-013) and conducted in accordance with the tenets of the Declaration of Helsinki. Informed consent was not required because only fully anonymized data were used. An information leaflet was posted at the hospital to inform patients and their guardians about the general use of clinical data for research purposes.

We included patients based on the following criteria: successful resuscitation after OHCA with subsequent admission to the pediatric intensive care unit (PICU); age ≤18 years; a pre-arrest pediatric cerebral performance category (PCPC) score of 1–3; and receipt of TTM in the PICU. TTM was applied according to a standardized institutional protocol. All patients who met these prespecified eligibility criteria had complete data for the variables required to calculate the rCAST score; therefore, no patients were excluded because of missing rCAST data and no imputation was performed. The first-line method involved surface cooling using a circulating water system with hydrogel pads combined with a computerized temperature control unit. When this system was unavailable, alternative approaches, such as antipyretic and analgesic medications, conventional cooling methods, or extracorporeal circulation devices, were employed. The core temperature was primarily measured using an esophageal probe; bladder or rectal probes were used when necessary. The target temperature (typically approximately 36°C) was maintained for at least 24 h in accordance with international guidelines. TTM was initiated as early as possible, ideally within 6 h after ROSC, followed by controlled normothermia maintained until 72 h after ROSC. In certain cases, modifications were made at the discretion of the attending physician, such as extending the duration of TTM or selecting alternative cooling strategies, depending on device availability and patient-specific factors. The administration of sedatives and analgesics was determined by the attending intensivist in line with the institutional protocols, whereas neuromuscular blocking agents were used when necessary to control shivering or ventilator–patient dyssynchrony.

### Outcome measures

Patients were classified into three severity groups based on their rCAST scores: low, 0–5.5; moderate, 6.0–14.0; and high, 14.5–18.5.^5^, ^6^ The primary outcome was neurological status at the time of PICU discharge, categorized as favorable (no decline in the PCPC score) or unfavorable (decline in the PCPC score or death). As a sensitivity analysis, neurological outcome was additionally classified using an absolute PCPC threshold, with PCPC scores of 1–2 defined as favorable and scores of 3–6 as unfavorable at PICU discharge, consistent with the outcome definition used in the previous pediatric rCAST study. The PCPC and rCAST scores were independently assessed by two intensivists (YS and SN). In the case of discrepancies, a consensus meeting was held to reach an agreement. Secondary outcomes assessed at PICU discharge included PICU length of stay and the number of ventilator-free days within the first 28 days after PICU admission (28-VFDs). The 28-VFDs were defined as the number of days alive and free from mechanical ventilation during the first 28 days following PICU admission and were calculated as 28 minus the number of days of mechanical ventilation. Patients who required mechanical ventilation for 28 days or longer were assigned a 28-VFD value of 0.^12^

### Data collection

All data were reviewed and extracted from the SCMC electronic medical record system. Collected variables included patient characteristics (age, sex, pre-existing illnesses, including those identified through etiological investigations, Pediatric Index of Mortality 2, and pre-arrest PCPC score); OHCA characteristics (etiology of cardiac arrest classified according to the final diagnosis documented by the primary clinical team, initial rhythm, whether the arrest was witnessed or unwitnessed, bystander cardiopulmonary resuscitation, time to ROSC, and initial blood pH, blood lactate concentration, and GCS motor score); post-OHCA characteristics (route of PICU admission, 28-VFDs, maximal vasoactive inotrope score within 24 h (VIS 24 h), extracorporeal membrane oxygenation [ECMO], and tracheostomy); and outcomes (PCPC score at PICU discharge and PICU length of stay).

The GCS motor score used for rCAST calculation was assessed at the time of ROSC, in accordance with the original rCAST definition, and was categorized as M1 or ≥M2 based on the documented motor response to stimulation.

The maximal VIS at 24 h was defined as the highest dosing rates of vasoactive and inotropic medications (μg/kg/min or IU/kg/min) administered during the first 24 h after PICU admission. These data were retrieved via PICU database queries and calculated using the following formula: VIS = dopamine dose (μg/kg/min) + dobutamine dose (μg/kg/min) + 100 × epinephrine dose (μg/kg/min) + 50 × levosimendan dose (μg/kg/min) + 10 × milrinone dose (μg/kg/min) + 10,000 × vasopressin dose (units/kg/min) + 100 × norepinephrine dose (μg/kg/min).^13^

To minimize information bias, data were extracted using a standardized data collection form. Two investigators (YS and SN) independently reviewed key variables, including PCPC and rCAST scores, to ensure consistency.

### Statistical analysis

Because pediatric OHCA is uncommon and this retrospective study covered a predefined study period, no formal a priori sample-size calculation was performed. All consecutive patients who met the prespecified eligibility criteria during the study period were included. Patient, OHCA, and outcome characteristics were summarized using descriptive statistics and compared across the three rCAST severity groups. Continuous nonparametric variables were analyzed using the Kruskal–Wallis test and are presented as medians with interquartile ranges (IQRs). Categorical variables were compared using Fisher’s exact test and are reported as counts (*n*) and percentages (%).

The discriminative performance of the rCAST score for unfavorable neurological outcome and in-hospital mortality was assessed using receiver operating characteristic (ROC) curves and the area under the curve (AUC), with 95% confidence intervals (CIs) calculated using the DeLong method. The discriminative performance of the rCAST score was also evaluated in the sensitivity analysis using the absolute PCPC 1–2 versus 3–6 outcome definition. As an exploratory assessment of calibration and overall model performance, cohort-specific logistic regression models using the rCAST score as the sole predictor were fitted separately for unfavorable neurological outcome and in-hospital mortality. Apparent overall performance was summarized using the Brier score. Internal validation was performed using 500 bootstrap resamples to assess optimism and the internal stability of the fitted score-to-outcome relationships, from which optimism-corrected C-indices and calibration slopes were obtained. An ideal calibration slope is 1.0, with values <1.0 indicating overfitting (shrinkage). All statistical analyses were conducted using R version 4.2.2 (R Foundation for Statistical Computing, Vienna, Austria), with the pROC and rms packages. Statistical significance was defined as a two-sided *P*-value < 0.05.

## Results

Among 5456 total PICU admissions during the study period, 91 patients experienced OHCA and were diagnosed with PCAS. Sixteen patients were excluded because of pre-arrest PCPC scores of 4–6; none were excluded because of missing data required for calculation of the rCAST score. The remaining 75 patients were included and stratified by rCAST score into the low- (*n* = 17), moderate- (*n* = 11), and high-severity (*n* = 47) groups (Fig. 1). Patient characteristics are summarized in Table 1; all outcomes and estimates are presented as unadjusted values according to rCAST-defined severity groups. The overall median age was 17 months (IQR, 4–72). The median age was significantly lower in the high-severity group (10 months [IQR, 4–27]) than in the low-severity group (72 months [IQR, 23–171]; *P* = 0.002). No significant differences were observed in sex distribution (*P* = 0.33) or route of PICU admission (*P* = 0.32). Premorbid neurodevelopmental status was predominantly a PCPC score 1 overall and particularly common in the high-severity group, whereas PCPC scores of 2–3 were significantly more frequent in the low- and moderate-severity groups (*P* = 0.007). Most patients had no pre-existing conditions (51/75, 68%). Cardiac disease was more prevalent in the low- (7/17, 41%) and moderate-severity groups (4/11, 36%) than in the high-severity group (2/47, 4%; *P* < 0.001). Chromosomal or genetic disorders and multiple congenital anomalies were distributed across all groups without significant differences (*P* = 0.07).

**Fig. 1:**
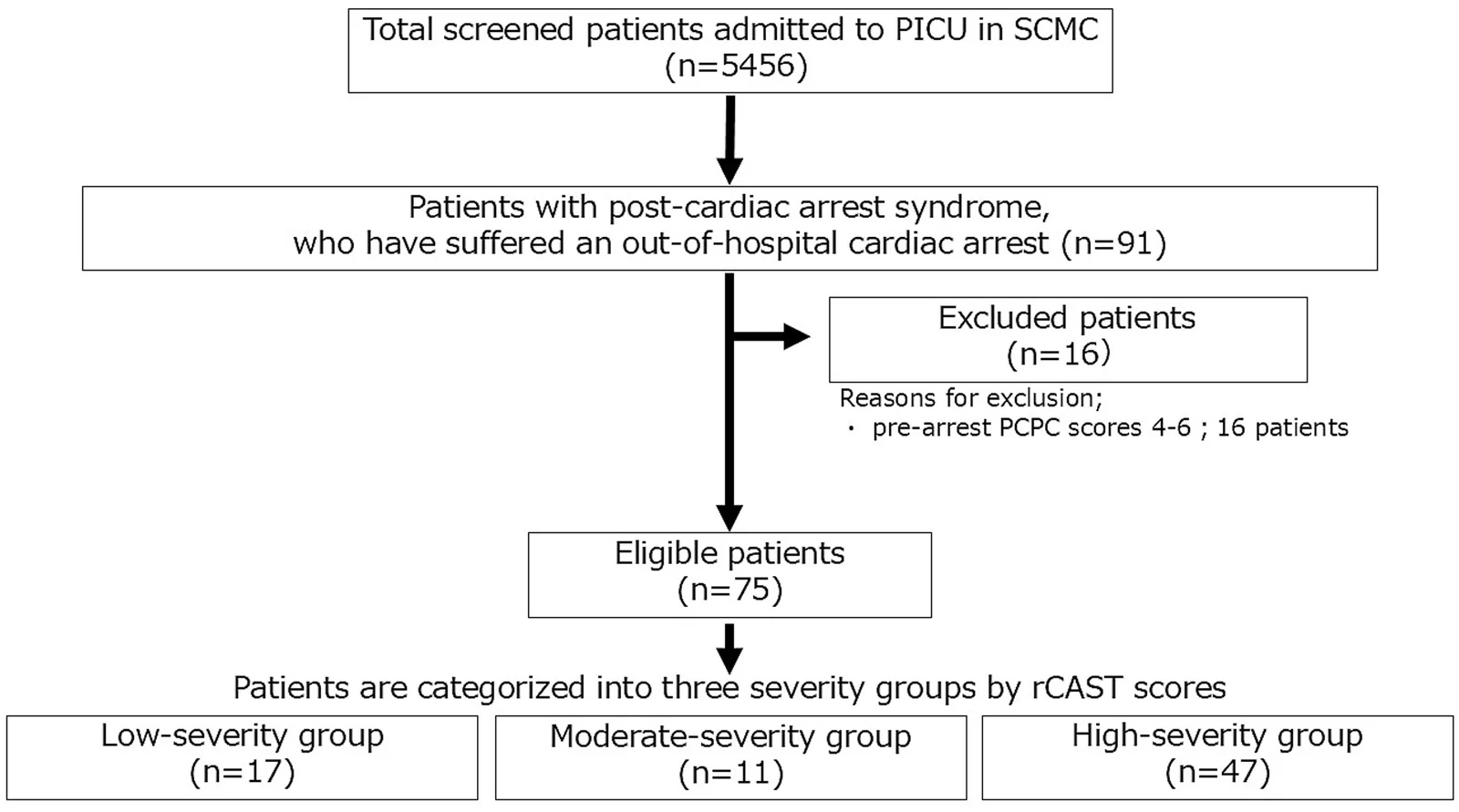
**Flowchart of the study patients**. Among 5456 PICU admissions, 91 cases of PCAS following OHCA were identified. Sixteen patients were excluded because of pre-arrest PCPC scores of 4–6; none were excluded because of missing data required for calculation of the rCAST score. The remaining 75 patients were included and classified into three severity groups based on the rCAST score. PICU, pediatric intensive care unit; SCMC, Saitama Children's Medical Center; PCPC, pediatric cerebral performance category; rCAST, revised post-Cardiac Arrest Syndrome for Therapeutic hypothermia.

**Table 1:** Comparison of patient characteristics for the total cohort and across severity groups.

	**Total**	**Low-severity group**	**Moderate-severity group**	**High-severity group**	***P*-value**
	**(*n* = 75)**	**(*n* = 17)**	**(*n* = 11)**	**(*n* = 47)**	
Age (months) [IQR]	17 [4–72]	72 [23–171]	29 [1.5–146]	10 [4–27]	0.002
Sex (male, %)	43 (57)	10 (59)	4 (36)	29 (62)	0.33
Route of PICU admission (direct, %)	37 (49)	11 (65)	4 (36)	22 (47)	0.319
PIM-2 (%), median [IQR]	84.9 [30.8–97.9]	7.4 [5.5–28.1]	35.8 [23.25–80.1]	96.3 [85–98.85]	<0.001

**Premorbid neurodevelopment**					
PCPC 1, *n* (%)	68 (91)	14 (82)	8 (72)	46 (98)	0.007
PCPC 2, *n* (%)	0 (0)	0 (0)	0 (0)	0 (0)	1
PCPC 3, *n* (%)	7 (9)	3 (18)	3 (27)	1 (2)	0.007

**Pre-existing conditions**					
None, *n* (%)	51 (68)	7 (41)	4 (36)	40 (85)	<0.001
Cardiac, *n* (%)	13 (17)	7 (41)	4 (36)	2 (4)	<0.001
Respiratory, *n* (%)	3 (4)	3 (18)	0	0	0.013
Neurological, *n* (%)	7 (9)	3 (18)	2 (18)	2 (4)	0.1
Chromosomal/genetic disorders and multiple congenital anomalies, *n* (%)	5 (7)	2 (12)	2 (18)	1 (2)	0.071

PICU, pediatric intensive care unit; PIM-2, pediatric index of mortality-2; IQR, interquartile range; PCPC, pediatric cerebral performance category.

OHCA characteristics are summarized in Table 2. The most common etiologies of cardiac arrest were asphyxia (15/75, 20%), sudden infant death syndrome (SIDS; 11/75, 15%), cardiogenic shock (9/75, 12%), drowning (9/75, 12%), and ventricular fibrillation (Vf; 7/75, 9%). Significant group differences were observed for SIDS, which occurred exclusively in the high-severity group (11/47, 23%; *P* = 0.02), and for Vf, which was more frequent in the low- (5/17, 29%) and moderate-severity groups (2/11, 18%) but absent in the high-severity group (0/47; *P* < 0.001). The most common initial rhythm was asystole (39/75, 52%), followed by pulseless electrical activity (14/75, 19%), Vf (7/75, 9%), bradycardia (1/75, 1%), and unknown rhythm (14/75, 19%). Significant differences across severity groups were observed for asystole, Vf, and unknown rhythms (*P* < 0.001 for asystole and unknown rhythms; *P* = 0.016 for Vf). Unwitnessed arrests occurred in 20/75 (27%) cases and were more frequent in the higher-severity groups (low: 1/17 [6%]; moderate: 2/11 [18%]; high: 17/47 [36%]; *P* = 0.034). The median time to ROSC was 50 min (IQR, 13.5–100.5), and the median no-flow time was 10 min (IQR, 0–41.5), both of which were significantly longer in higher severity groups (*P* < 0.001). Acidemia (pH < 7.0) was present in 55/75 (73%) cases, and the median blood lactate concentration was 13.79 mmol/L (IQR, 1.4–24), with significant differences across groups (*P* < 0.001). GCS motor scores were M1 in 51/75 (68%) cases and ≥M2 in 24/75 (32%), with marked differences by severity (*P* < 0.001).

**Table 2:** Comparison of OHCA characteristics for the total cohort and across severity groups.

	**Total**	**Low-severity group**	**Moderate-severity group**	**High-severity group**	***P*-value**
	**(*n* = 75)**	**(*n* = 17)**	**(*n* = 11)**	**(*n* = 47)**	
**Cause of cardiac arrest**					
Asphyxia, *n* (%)	15 (20)	2 (12)	1 (9)	12 (26)	0.40
SIDS, *n* (%)	11 (15)	0	0	11 (23)	0.02
Drowning, *n* (%)	9 (12)	3 (18)	1 (9)	5 (11)	0.78
Cardiogenic shock, *n* (%)	9 (12)	3 (18)	2 (18)	4 (9)	0.39
Vf, *n* (%)	7 (9)	5 (29)	2 (18)	0	<0.001
Child abuse syndrome, *n* (%)	6 (8)	0	1 (9)	5 (11)	0.46
Sepsis, *n* (%)	4 (5)	0	2 (18)	2 (4)	0.13
Strangulation, *n* (%)	4 (5)	3 (18)	0	1 (2)	0.05
Acute encephalopathy, *n* (%)	3 (4)	1 (6)	0	2 (4)	1
Cerebellar hemorrhage, *n* (%)	3 (4)	0	0	3 (6)	0.73
Carbon monoxide poisoning, *n* (%)	2 (3)	0	1 (9)	1 (2)	0.32
Status epilepticus, *n* (%)	1 (1)	0	1 (9)	0	0.15
Traffic trauma, *n* (%)	1 (1)	0	0	1 (2)	1

**Initial rhythm**					
Asystole, *n* (%)	39 (52)	1 (6)	1 (9)	37 (79)	<0.001
PEA, *n* (%)	14 (19)	2 (12)	4 (36)	8 (17)	0.29
Vf, *n* (%)	7 (9)	4 (24)	2 (18)	1 (2)	0.02
Bradycardia, *n* (%)	1 (1)	0	0	1 (2)	1
Unknown, *n* (%)	14 (19)	10 (59)	4 (36)	0	<0.001
Unwitnessed arrest, *n* (%)	20 (27)	1 (6)	2 (18)	17 (36)	0.03
Bystander cardiopulmonary resuscitation (CPR), *n* (%)	55 (73)	16 (94)	9 (82)	30 (64)	0.03
Time to ROSC (min), median [IQR]	50 [13.5–100.5]	8 [3–12]	15 [8.5–18.5]	83 [57–132]	<0.001
No-flow time (min), median [IQR]	10 [0–41.5]	0 [0–5]	0 [0–7.5]	30 [10–75.5]	<0.001

**Blood pH**					
<7.0, *n* (%)	55 (73)	0	9 (82)	46 (98)	<0.001
≥7.0, *n* (%)	20 (27)	17 (100)	2 (18)	1 (2)	<0.001
Blood lactate level (mmol/L), median [IQR]	13.79 [1.4–24]	5.2 [2.3–5.9]	11.3 [8.2–13.3]	15.7 [13.4–18.0]	<0.001

**Motor score on the GCS**					
M1, *n* (%)	51 (68)	1 (6)	3 (27)	47 (100)	<0.001

OHCA, out-of-hospital cardiac arrest; CPR, Cardiopulmonary resuscitation; ROSC, Return of Spontaneous Circulation; GCS, Glasgow Coma Scale; IQR, interquartile range; SIDS, Sudden Infant Death Syndrome; Vf, ventricular fibrillation; PEA, pulseless electrical activity.

Clinical outcomes are summarized in Table 3. The overall median maximum VIS within 24 h was 5 (IQR, 0–27.5) and significantly increased with severity (low: 0 [0–0]; moderate: 4 [0–10]; high: 12 [1.5–37]; *P* < 0.001). Median 28 ventilator-free days (28-VFDs) decreased significantly with increasing severity (overall: 0 [0–20.5]; low: 28 [22–28]; moderate: 19 [6–23.5]; high: 0 [0–0]; *P* < 0.001). ECMO was required in 4/75 (5%) patients: 2 (12%) in the low-severity group, 2 (18%) in the moderate-severity group, and none in the high-severity group (*P* = 0.017). Tracheostomy was performed in 5/75 (7%) patients, with no significant differences between groups (*P* = 1.0). The median PICU length of stay was 5 days (IQR, 2–14), with the longest stay observed in the moderate-severity group (15 [4–35] days; *P* = 0.09).

**Table 3:** Summary of clinical outcomes for the total cohort and comparisons across severity groups.

**Characteristics**	**Total**	**Low-severity group**	**Moderate-severity group**	**High-severity group**	***P*-value**
	**(*n* = 75)**	**(*n* = 17)**	**(*n* = 11)**	**(*n* = 47)**	
**Interventions during PICU stay**					
Max VIS 24 h, median [IQR]	5 [0–27.5]	0 [0–0]	4 [0–10]	12 [1.5–37]	<0.001
28-VFDs (days), median [IQR]	0 [0–20.5]	28 [22–28]	19 [6–23.5]	0 [0–0]	<0.001
ECMO, *n* (%)	4 (5)	2 (12)	2 (18)	0 (0)	0.017
Tracheostomy, *n* (%)	5 (7)	1 (6)	1 (9)	3 (6)	1

**Outcome**					
PICU stay time (days), median [IQR]	5 [2–14]	6 [4–12]	15 [4–35]	5 [2–12]	0.09
Favorable neurological outcome*, *n* (%)	21 (28)	13 (76)	8 (73)	0 (0)	<0.001
Unfavorable neurological outcome**, *n* (%)	54 (72)	4 (24)	3 (27)	47 (100)	<0.001
In-hospital mortality, *n* (%)	45 (60)	1 (6)	3 (27)	41 (87)	<0.001

PICU, pediatric intensive care unit; VIS 24 h, vasoactive inotrope score within 24 h; ECMO, extracorporeal membrane oxygenation; IQR, interquartile ranges; 28-VFDs, ventilator-free days within the first 28 days.

* Favorable neurological outcome was defined as no decline in the pediatric cerebral performance category (PCPC) score at PICU discharge compared with baseline.

** Unfavorable neurological outcome was defined as a decline in PCPC score or death at PICU discharge.

Favorable neurological outcomes at PICU discharge were observed only in the low- (13/17, 76%) and moderate-severity groups (8/11, 73%), whereas no patients in the high-severity group achieved favorable outcomes (*P* < 0.001). Overall, unfavorable neurological outcomes occurred in 54/75 (72%) patients, including 4/17 (24%) in the low-severity group, indicating that unfavorable outcomes may occur even among patients with low rCAST scores. In-hospital mortality occurred in 45/75 (60%) patients, with one death in the low-severity group (1/17, 6%) and significantly higher mortality rates in the moderate- (3/11, 27%) and high-severity groups (41/47, 87%; *P* < 0.001).

A ROC curve analysis was performed to assess the predictive accuracy of rCAST scores for neurological outcomes and mortality (Fig. 2). The AUC was 0.947 (95% CI, 0.894–1.000) for unfavorable neurological outcome and 0.857 (95% CI, 0.750–0.964) for mortality. In the sensitivity analysis using an absolute PCPC threshold (PCPC 1–2 vs. 3–6), the rCAST score showed similar discrimination for unfavorable neurological outcome, with an AUC of 0.924 (95% CI, 0.863–0.986). Regarding overall model performance, the apparent Brier scores were 0.086 for unfavorable neurological outcome and 0.106 for mortality. After internal validation using 500 bootstrap resamples, the optimism-corrected C-indices were 0.946 and 0.858, respectively, with corresponding optimism-corrected calibration slopes of 0.952 and 0.988 (Supplementary Fig. S1).

**Fig. 2:**
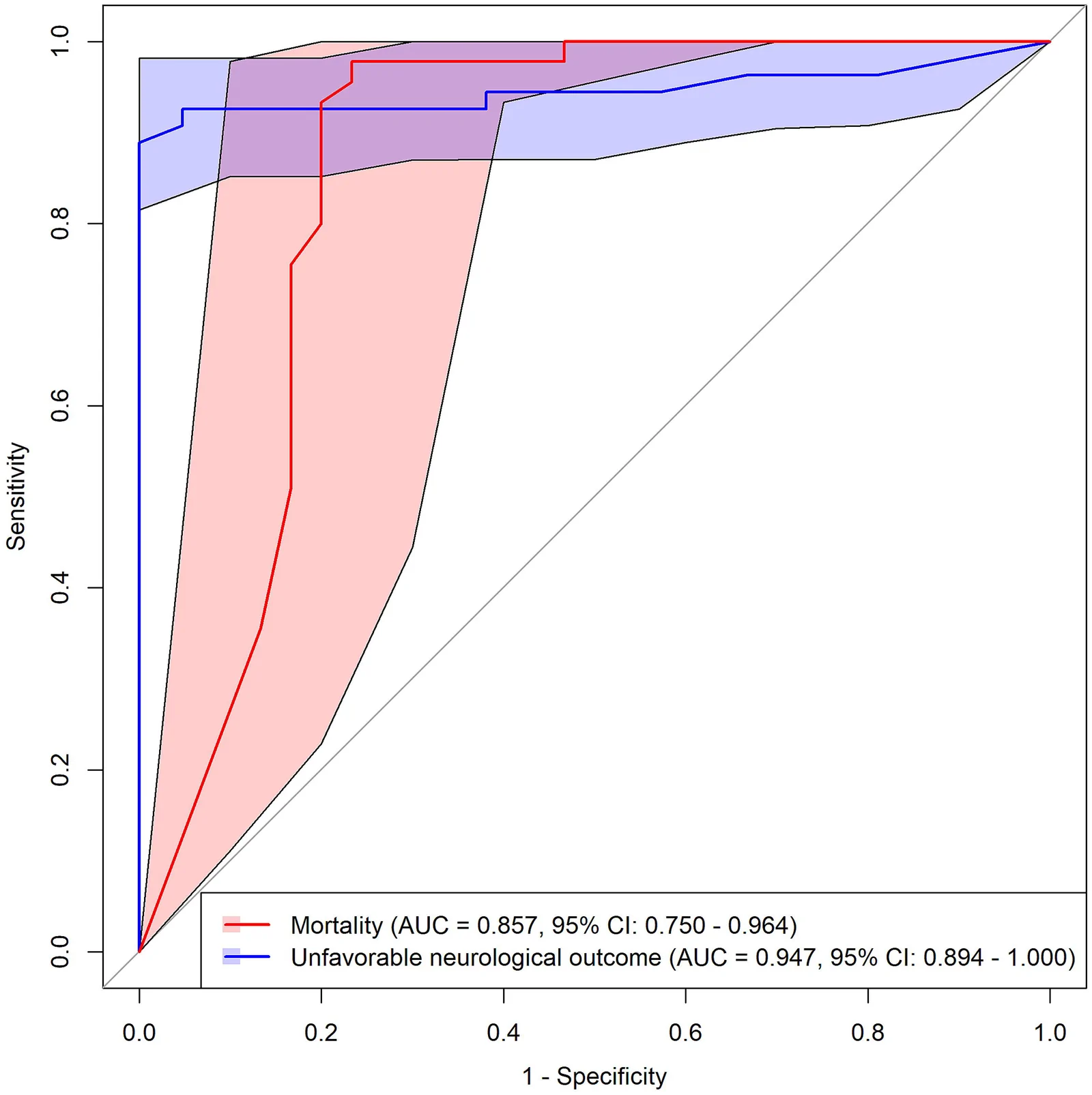
**Receiver operating characteristic curves of the rCAST score for unfavorable neurological outcome and in-hospital mortality**. The AUC was 0.947 (95% CI, 0.894–1.000) for unfavorable neurological outcome and 0.857 (95% CI, 0.750–0.964) for in-hospital mortality. CI, confidence interval; AUC, area under the curve; ROC, receiver operating characteristic; rCAST, revised post-Cardiac Arrest Syndrome for Therapeutic hypothermia.

## Discussion

We externally validated rCAST scores in infants and young children with PCAS following OHCA, and the results demonstrated good discriminative ability for neurological prognosis and in-hospital mortality. Infants and young children were more frequently classified in the moderate- to high-severity groups than in the low-severity group. Although a subset of individuals in the low-severity group experienced unfavorable neurological outcomes, all individuals in the high-severity group had uniformly poor outcomes. The distributions of age, sex, etiology, and outcomes were largely consistent with those reported in national epidemiological studies.^7^

Our results support the utility of the rCAST score in predicting neurological outcomes in pediatric patients with PCAS following OHCA, consistent with a previous pediatric study.^6^ The discriminative ability of the rCAST score for predicting unfavorable neurological outcomes was similarly high in both studies (AUC 0.947 vs. 0.95). All individuals in the high-severity group had unfavorable neurological outcomes. For mortality, the AUC observed in our cohort was numerically higher than that reported in the previous pediatric study (0.857 vs. 0.68)^6^ and was within the range reported in adult rCAST studies, in which AUCs ≥0.80 have been reported.^5^ However, this difference should be interpreted descriptively because no formal between-study comparison was performed and differences in case mix and study populations may have influenced the observed estimates. More broadly, prognostication remains challenging in pediatric patients with coma or disorders of consciousness because their natural history and long-term outcomes remain incompletely characterized and widely applicable prognostic tools are limited.^14^, ^15^ Prognosis following comatose states in children after non-traumatic brain injury is generally poor, with most remaining in a vegetative state (65%) or dying (22%) within 1 year.^16^ However, favorable outcomes have been reported in a subset of cases,^17^, ^18^, ^19^, ^20^ and advances in resuscitation practices have gradually improved survival among children with disorders of consciousness.^19^ Accordingly, at our institution, individuals with severe and irreversible brain dysfunction who meet criteria for brain death are managed using a palliative approach. Life-sustaining treatments are withheld or withdrawn after comprehensive discussions involving a multidisciplinary team and the family, allowing death to occur naturally.

The rCAST score retains its discriminative ability for neurological outcomes even in this younger age group. A prior pediatric rCAST study focused on relatively older children,^6^ and extending its applicability to younger patients offers two important advantages. First, pediatric OHCA predominantly occurs in infants and toddlers,^7^, ^8^, ^9^, ^10^ making this evaluation more representative of real-world clinical practice. Second, previous studies have reported poorer outcomes in infants and younger children,^20^, ^21^ underscoring the need to determine whether the rCAST score can reliably assess neurological prognosis in this population. Our results highlight its clinical value in infants and young children. In our study, mortality was higher and unfavorable neurological outcomes occurred even in the low-severity group (4/17, 24%), whereas the previous study reported favorable outcomes in all individuals classified as low severity.^6^ This discrepancy may be attributable to the inclusion of infants and younger children in the present cohort. It is plausible that injury to the immature brain leads to profound yet less efficient functional reorganization, and that certain etiologies, such as encephalitis or stroke, predispose individuals to secondary brain injury.^22^ The immature brain is particularly vulnerable to excitotoxicity, as a high density of glutamate receptors during active synaptogenesis can trigger excessive calcium influx and apoptosis even after seemingly mild ischemic insults.^23^ In addition, the age range of this cohort overlaps with a period of rapid myelination, during which premyelinating oligodendrocytes are highly susceptible to ischemia and inflammation, increasing the risk of secondary white matter injury.^24^ Poor-outcome cases in the low-severity group were classified within these categories. Thus, even individuals classified as having low severity by the rCAST score may remain at risk, and age-specific vulnerabilities may amplify secondary brain injury cascades beyond what is captured by initial severity scoring.

The prognostic value of the rCAST score in this infant-predominant cohort is particularly noteworthy when considered alongside other established pediatric predictive tools. For example, although the Brain Death After Cardiac Arrest score has demonstrated good performance in older children, its predictive accuracy declines substantially in infants,^25^ who account for the highest incidence of pediatric OHCA. Similarly, the Pediatric Termination of Resuscitation rule, while clinically useful, was designed to guide prehospital termination decisions rather than predict post-cardiac arrest outcomes in survivors^26^ and therefore provides limited insight into neurological prognosis. Our findings also complement age-stratified risk models derived from the Cardiac Arrest Registry to Enhance Survival data, which emphasize the unique challenges of prognostication in infants.^27^ By providing additional data on the discriminative performance of the rCAST score in this vulnerable population, this study addresses an important gap in existing tools and offers a new perspective for guiding clinical decision-making during the post-resuscitation period in infants and young children.

This study has some limitations. First, this was a single-center study conducted in a limited geographic setting with a relatively small sample size, which limited the precision of the performance estimates, particularly for mortality, as reflected by the width of the 95% confidence interval. Second, the analysis was restricted to patients who received TTM, which may have introduced selection bias and may limit the generalizability of our findings to pediatric patients with PCAS who do not undergo TTM. Although this criterion is consistent with the population in which the rCAST score was originally developed, the applicability of our findings to patients managed without TTM remains uncertain. Third, although the rCAST uses a simple dichotomization of the GCS motor score (M1 vs. ≥M2), its assessment in infants and young children may still be influenced by developmental stage and clinical factors surrounding resuscitation. Fourth, unlike the previous pediatric rCAST study,^6^ which included only patients with pre-arrest PCPC scores of 1–2, our cohort included patients with scores of 1–3. This broader criterion was intended to reflect the clinical heterogeneity of pediatric patients with PCAS, including children with pre-existing developmental or neurological impairment who retained meaningful premorbid function. Because PCPC 3 represents moderate disability without the severe functional dependence represented by PCPC 4–6, preservation of the individual pre-arrest PCPC status was considered clinically relevant. Importantly, a sensitivity analysis using the conventional absolute PCPC threshold of 1–2 versus 3–6 yielded similar discrimination, suggesting that the main findings were not substantially affected by the choice of neurological outcome definition. Fifth, neurological outcomes were assessed at PICU discharge and may not fully reflect long-term prognosis. Sixth, although our findings and previous studies^5^, ^6^ support the discriminative ability of the rCAST score for neurological outcomes, its calibration remains uncertain, as emphasized by Alba et al.^28^ In bootstrap internal validation of models using the rCAST score as the sole predictor, the optimism-corrected C-indices were 0.858 for mortality and 0.946 for unfavorable neurological outcome, with corresponding optimism-corrected calibration slopes of 0.988 and 0.952, respectively (Supplementary Fig. S1), suggesting limited optimism. However, the calibration curves showed greater departures from ideal calibration in the intermediate-risk range, particularly for unfavorable neurological outcome, likely reflecting, at least in part, the modest sample size and sparse distribution of patients within this range. Because miscalibration may lead to over- or underestimation of risk and potentially affect clinical decision-making or communication with families, these findings should be interpreted cautiously. Larger multicenter studies are warranted to further evaluate the calibration and overall predictive performance of the rCAST score in pediatric populations. Seventh, although the overall institutional strategy for post-cardiac arrest care, including TTM, remained broadly consistent during the study period, incremental changes in clinical practice associated with evolving guidelines and evidence cannot be excluded.^29^ Such temporal changes may have influenced patient management and outcomes.

## Conclusions

The rCAST score showed good discrimination for unfavorable neurological outcomes in pediatric patients with PCAS following OHCA, including infants and young children. These findings support its potential utility for risk stratification, while the occurrence of unfavorable outcomes in some low-score patients warrants cautious interpretation and further validation.

## Availability of data and materials

The datasets used and analyzed in this study are available from the corresponding author upon reasonable request.

## Declaration of generative AI and AI-assisted technologies in the manuscript preparation process

During the preparation of this work, the authors used ChatGPT® in order to refine the language and align the style with scholarly journal standards. After using this tool, the authors reviewed and edited the content as needed and take full responsibility for the content of the published article.

## CRediT authorship contribution statement

**Yuta Suenaga:** Writing – review & editing, Writing – original draft, Visualization, Validation, Project administration, Methodology, Investigation, Funding acquisition, Formal analysis, Data curation, Conceptualization. **Satoshi Nakano:** Writing – review & editing, Validation, Methodology, Investigation, Data curation, Conceptualization. **Takehiro Nitsu:** Writing – review & editing. **Ikuya Ueta:** Writing – review & editing, Supervision.

## Funding

This work was supported by Saitama Children’s Medical Center (Grant Number 2025-A5).

## Declaration of competing interest

The authors declare that they have no conflicts of interest.

## Figures and Tables

**Supplementary Fig. S1:**
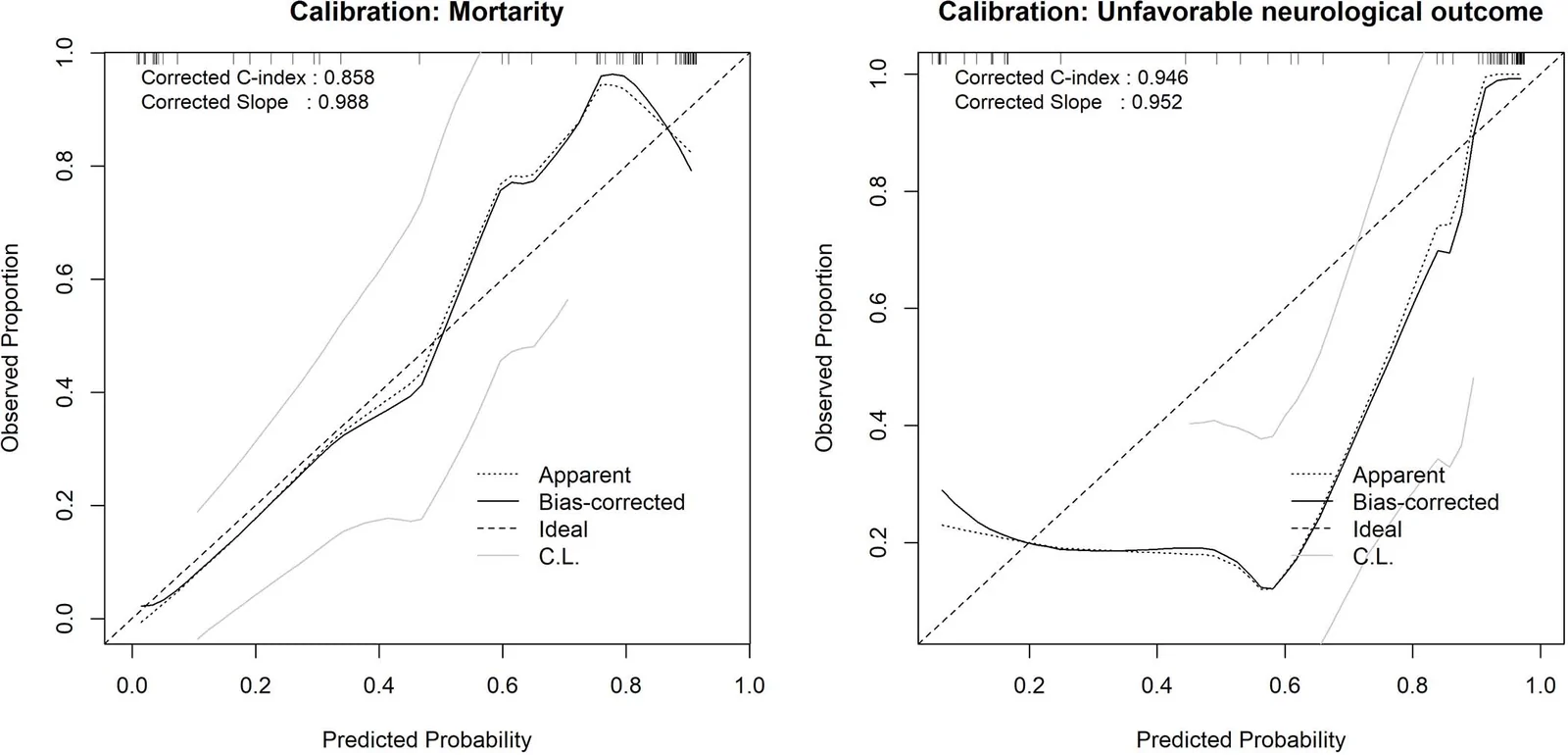
**Bootstrap-corrected calibration of cohort-specific models using the rCAST score as the sole predictor.** Apparent Brier scores were 0.086 for unfavorable neurological outcome and 0.106 for in-hospital mortality. Internal validation was performed using 500 bootstrap resamples. The optimism-corrected C-indices were 0.946 and 0.858, with corresponding calibration slopes of 0.952 and 0.988, respectively. These analyses assessed optimism and the internal stability of the fitted score-to-outcome relationships within the present cohort.
